# Efficacy of ultrasound-guided obturator nerve block in transurethral surgery

**DOI:** 10.4103/1658-354X.76507

**Published:** 2011

**Authors:** Ahmed Thallaj, Dany Rabah

**Affiliations:** *Assistant Professor of Anesthesia, Urologist, College of Medicine, King Saud University, Riyadh, KSA*; 1 *Associate Professor, Urologist, College of Medicine, King Saud University, Riyadh, KSA*

**Keywords:** *Obturator nerve block*, *transurethral resection of prostate*, *ultrasound guided*

## Abstract

**Background:**

During transurethral resection surgery (TUR), accidental stimulation of the obturator nerve can cause violent adductor contraction, leading to serious intraoperative complications. General anesthesia with muscle relaxation is currently the preferred technique for TUR surgery. Spinal anesthesia combined with obturator nerve block has also been used for TUR surgery in geriatric population. Blind, anatomical methods for identifying the obturator nerve are often unsatisfactory. Therefore, we conducted this prospective study to validate the efficacy of ultrasound-guided obturator nerve block (USONB) during TUR procedures.

**Methods:**

Eighteen male patients undergoing TURP surgery under spinal anesthesia were included in the study. Bilateral USONB with maximum 20 ml of 1% lidocaine per patient was performed. An independent observer was present to monitor any adduction movements during the operation and to record patient and surgeon satisfactions.

**Results:**

In all patients, obturator nerve was visualized from the first attempt, requiring an average of 4.3 min for blocking of each side. USONB was successful (97.2%) in preventing an adductor spasm in all except one patient. Patient’s and surgeon’s satisfaction were appropriate. In all patients, adductor muscle strength recovered fully within 2 h following the surgical procedure.

**Conclusions:**

USONB is safe and effective during TUR surgery. It provides optimal intra-and postoperative conditions.

## INTRODUCTION

The obturator nerve originates from the second, third, and fourth lumbar segments and descends into the pelvis. It passes over the psoas major muscle fibers and eventually enters the medial aspect of the thigh where it emerges out and innervates the adductor muscles. Accidental stimulation of the obturator nerve is a serious concern during various surgical operations, and in particular, during TUR procedures, due to the passage of the nerve close to the bladder wall. Inadvertent adductor muscle spasm during these procedures can cause complications such as bladder perforation, excessive bleeding and/or discontinuation of the operation. Administration of general anesthesia (GA) with muscle relaxants is currently the reliable way to prevent the adductor spasm.[1] During transurethral operations it is preferable to use spinal anesthesia supplemented with selective, bilateral obturator nerve blocks since alternative methods to prevent obturator nerve stimulation such as reducing the electro-coagulation voltage, incomplete bladder filling or resections of smaller ships are not effective and may lead to incomplete resection of bladder tumors.[2] Several options have been proposed for obturator nerve block. The “3-in-1” block proposed by Winnie for simultaneously blocking the femoral, femoral cutaneous and the obturator nerves by injection into the femoral nerve sheath has been controversial regarding its efficacy for an obturator nerve block.[3] Traditional methods rely on anatomical landmarks for establishing obturator nerve block and may involve using a nerve stimulator.[4] In the literature, there is only one report on ultrasound-guided obturator nerve block (USONB) for transurethral resection surgery (TUR) procedures where Fujiwara described 23 blocks for patients having TUR of a bladder tumor.[5] He stated that proof of a complete blockade of the obturator nerve could not be tested in his study due to motor blockade caused by spinal anesthesia. Therefore and in order to validate such technique, we conducted this prospective clinical observational study on a higher number of obturator nerve blocks to validate the efficacy of combined spinal anesthesia and bilateral USONB during TUR surgery.

## METHODS

**Figure 1:**
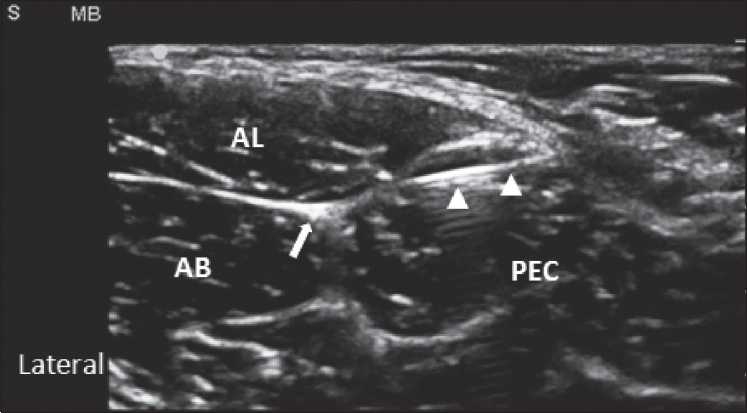
Ultrasound view of obturator nerve. PEC: pectineus muscle; AL: adductor longus muscle; AB: adductor brevis muscle; Arrow points at the anterior branch of the obturator nerve; triangles points at the needle

The study was undertaken after approval of the hospital ethics committee. Informed patient consent was obtained from all patients. Exclusion criteria included patient’s refusal, neurological deficit, and abnormal coagulation profile. A total of 18 consecutive male patients who were admitted to our hospital and recommended for TURP surgery were included in the study. After receiving spinal anesthesia with heavy bupivacaine 10 mg in the usual standard way, all 18 patients were subjected to bilateral USONB, thus allowing us to independently evaluate a total 36 blocks. Each patient was in supine position, with the thigh abducted and rotated externally. Using a two-dimensional (2D) ultrasound, linear probe 38 mm 6–13 MHz (SonoSite M-Turbo, SonoSite Inc., Bothell, WA, USA) under sterile conditions, the antero-medial aspect of the thigh was scanned. The probe was positioned on and was parallel to the inguinal ligament. A short access view allowed visualization of the pectineus muscle and the obturator nerve was embedded between the adductor longus and adductor brevis muscles. A 50 mm insulated needle was inserted parallel to the long axis of the probe and passed under vision to the anterior branch of the obturator nerve [Figure 1]. A solution of 1% lidocaine, maximum 10 ml on each side, was injected to selectively block the obturator nerve. The procedure was repeated on the other thigh. A transurethral resectoscope was used for electro-cauterization, applied in five separate areas on each lateral wall of the bladder in the region that usually overlies the pelvic segment of the obturator nerve. All TURP procedures were performed by the same surgeon. An independent observer was present to monitor the patients for any adduction movements during the entire procedure. Data collection included, time required for obturator nerve, identification by ultrasound, volume of lidocaine administered, time required for regaining adductor strength following the procedure, patient and surgeon satisfactions, and adverse events if any. The sample size calculation was based on the assumption that the patients undergoing TUR will not show adductor spasm in 97% of the patients. Assuming a power of 90%, a level of significance of 5%, it was estimated that 18 patients would be required with 36 USONB procedures.

## RESULTS

Mean age of the patients was 71.1 year (S.D. ± 14.0 year). All patients underwent TURP surgery during which they were subjected to bilateral USONB. Right and left blocks were performed independently to have a total of 36 blocks. The obturator nerve could be identified in the first attempt in all cases. The average time taken to visualize the obturator nerve was 4.3 min (S.D. ± 0.8 min). The mean volume of the local anesthetic drug used was 9.0 ml (S.D. ± 1.4 ml). There were no adverse events. Out of the 36 obturator nerve blocks, only one failed to prevent adductor muscle spasm, marking a success rate of 97.2%. The reason for the failure in this case is unknown. Nevertheless, the intended operation was successfully completed in this patient. Patients and surgeon satisfactions were appropriate.

## DISCUSSION

An interadductor approach was recently discussed by Kakinohana *et al*.[6] This method involves insertion of a needle behind the adductor longus tendon combined with nerve stimulation to identify the obturator nerve. It proved to be faster and more successful than the traditional approach in terms of shortening the insertion–adduction contraction interval. In contrast, Tatlisen *et al*. have argued that the blind anatomical approach is satisfactory based on their experience with unilateral obturator nerve blocks performed during transurethral resection for bladder tumors.[7] Yet others have proposed a system of endoscopic surgery for TUR, advocating use of saline, an electro conductive solution, as the perfusate instead of the conventional nonelectro conductive solution. This method circumvents the need of ONB altogether.[8] However, a case of bladder perforation due to obturator nerve reflex using TUR in saline system under spinal anesthesia was described.[9] Successful blockade of the obturator nerve is desirable during TUR surgery. Given the complex anatomy in the pelvic region, the use of traditional methods that rely on anatomical landmarks to identify and block the obturator nerve carries the risk of failure or incomplete blockage. In one study on USONB involving 22 patients, the obturator nerve was correctly identified on the first attempt in 91% of the cases with decreased adductor strength in all patients.[10] Akkaya *et al*. demonstrated a new methodological approach based on a sono-anatomic study to visualize the obturator nerve. They reported patient’s satisfaction as 93%.[11] In our series, we found that the use of ultrasound technology was reliable in blocking the obturator nerve and we succeeded to locate the obturator nerve with decreased obturator reflex in 97.2% of the cases. In addition, we reported the same percentage for patient’s and surgeon satisfactions. USONB is particularly useful in surgeries for removal of tumors located on the lateral bladder wall as those tend to lie over the course of the pelvic segment of the obturator nerve. Except under specific pre-existing conditions such as obturator neuropathy, inguinal lymph adenoma or perineal infection, USONB would not be applicable during TUR surgery.

In the present study, we block only the anterior branch of the obturator nerve with up to 10 ml of local anesthetic, such a high volume will distribute between the aponurosis of the surrounding muscles to reach the main obturator nerve trunk.

In conclusion, we found that USONB was safe, fast and effective in TUR surgery. In all but one out of the 36 blocks administered, adductor spasm was prevented by an USONB. We believe that USONB is safe and effective during TUR surgery. It provides optimal intra and postoperative conditions. In this study, we validate the efficacy of the technique of USONB for TUR surgery, however, larger series; controlled studies are required to prove the efficacy of USONB not only for TUR, but also for other surgeries where ONB is indicated.
